# Genetic variations associated with telomere length affect the risk of gastric carcinoma

**DOI:** 10.1097/MD.0000000000020551

**Published:** 2020-06-05

**Authors:** Ma Lili, Fan Yuxiang, Han Zhongcheng, Su Ying, Chen Ru, Xu Rong, Liu Jiang

**Affiliations:** a Department of Oncology, People's Hospital of Xinjiang Uygur Autonomous Region; b The Second Department of Oncology, Traditional Chinese Medical Hospital of Xinjiang Uygur Autonomous Region (The Fourth Affiliated Hospital of Xinjiang Medical University), Urumqi, Xinjiang, China.

**Keywords:** case-control study, gastric carcinoma (GC), genetic variations, relative telomere length (RTL), single nucleotide polymorphism

## Abstract

This study aimed to further understand the role of relative telomere length (RTL) in susceptibility to gastric carcinoma (GC) and investigate the association between genetic polymorphisms in the telomere length related genes and GC risk.

RTL was measured using the real-time quantitative polymerase chain reaction from 1000 patients and 1100 healthy controls. Genotyping was performed using the Agena MassARRAY platform. The statistical analysis was performed using the chi-square/ Welch *T* tests, Mann–Whitney *U* test, and logistic regression analysis.

The association analysis of telomere length and GC showed that the RTL in the case group was shorter than in the controls, and the shorter RTL was associated with an increased risk of GC. The association analysis between telomere length related genes polymorphisms and genetic susceptibility to GC indicated that: In the allele models and genetic models, *TERT* (rs10069690, rs2242652 and rs2853676) and *TN1F1* (rs7708392 and rs10036748) were significantly associated with an increased risk of GC. In addition, the haplotype "G_rs10069690_C_rs2242652_” of *TERT* and the haplotype "G_rs7708392_T_rs10036748_” of *TNIP1* were associated with an increased risk of GC

Our results suggested that shorter RTL was associated with an increased risk of GC; The association analysis have identified that the *TERT* (rs10069690, rs2242652 and rs2853676) and *TN1P1* (rs7708392 and rs10036748) were associated with GC risk.

## Abstract 2

Supplemental Digital Content is available in the text

## 1 Introduction

Gastric carcinoma (GC), one of the most common human cancers, is a heterogeneous disease with high morbidity and mortality. Although the incidence has been declining in most parts of the world in the last decades, stomach carcinoma remains a prominent cancer worldwide and is responsible for over 1,000,000 new cases in 2018 and an estimated 783,000 deaths, making it the fifth most frequently diagnosed cancer and the third leading cause of cancer death.^[1]^ Smoking, high salt intake and a familial genetic component are also recognized as predisposing factors.^[2]^ Meanwhile, in recent years, many studies have shown that telomere length variation is strongly implicated in the process of carcinogenesis, although the current findings are still in debate.^[3,4]^ Additionally, various genetic and epigenetic alterations are associated with GC.^[5,6]^ Previously, genome-wide association analysis studies have identified many genes involved in gastric carcinogenesis and prognosis.^[7]^

Telomeres are specific structures located at the ends of eukaryotic chromosomes and are crucial in maintaining chromosome integrity and genomic stability.^[8]^ Telomere length progressively shortens during somatic-cell replication, because of the inability of DNA polymerase to fully replicate the 3′ end of the DNA.^[8]^ Telomere length is determined by the balance of processes that shorten and lengthen the telomere, thus leading to telomere variation in individuals at the same age.^[9]^ The maintenance of telomere length relies on the activity of telomerase, a reverse transcriptase complex that adds DNA sequence repeats (‘TTAGGG’ in all vertebrates) to the 3′ end of DNA strands in the telomere regions.^[10]^ The available evidence suggests that distinct cancer phenotypes are associated with both short and long telomere extremes. Telomeres also shorten in humans with age, and in the past decade, it has become clear that abnormally short telomeres can cause several age-related disease phenotypes.^[11]^ When telomeres become critically short, they activate a dnadeoxyribonucleic acid (DNA) damage response, which provokes cellular senescence or apoptosis.^[12]^ In the past 2 years, mutations that appear to lengthen telomeres have been linked to an increased risk of cancer. Unrestricted proliferation when telomeres are long would increase the likelihood of sustaining driver mutations that eventually promote a cancer clonal advantage and metastasis.^[13]^ Nowadays, little research has been done on telomere length and GC, the association between leukocyte telomere length and GC risk has not yet been assessed. Whether the incidence of GC is related to longer telomeres or shorter telomeres is worthy of systematic exploration.

The activity of telomerase can affect the telomere length, which in turn can affect the incidence of cancer or other diseases.^[14]^ However, telomerase activity and relative telomere length (RTL) can be directly or indirectly affected by many telomere related genes.^[15]^ Genetic association studies have indicated that polymorphisms in the telomerase reverse transcriptase-encoding gene *TERT* and related genes such as *TERC*, *MYNN*, *NAF1*, *TNIP1*, *STN1*, *ZNF208*, and *RTEL1* are associated with the variation of telomere length.^[16]^ However, there are few studies on telomere related genes and genetic susceptibility of GC. Therefore, it is necessary to explore the telomere related genes and the susceptibility of GC.

To identify the associations between telomere length and telomere related genes (*TERT*, *TERC*, *MYNN*, *NAF1*, *TNIP1*, *STN1*, *ZNF208*, and *RTEL1*) and GC risk in previous studies, we conduct a case-control study including 1000 cases and 1100 controls to further clarify their potential roles in GC risk in the Chinese population. To the best of our knowledge, this is the first epidemiological study to investigate the role of telomere length and telomere related genes in GC etiology.

## 2 Materials and methods

### 2.1 Participants and ethics statement

This case-control study involved 1000 GC patients and 1100 control subjects. All participants were conducted at the People's Hospital of Xinjiang Uygur Autonomous Region, and the healthy controls were the same race as the GC patients. Patients diagnosed with other types of cancer or underwent radiotherapy or chemotherapy were excluded. Healthy control subjects were recruited from the physical examination center at the same hospital. All control patients had no history of cancer. Additionally, healthy subjects were the same race as the GC patients and were age- and sex-matched with GC patients.

All participants were informed, both in writing and verbally, of the procedures and purpose of the study, and each participant signed informed consent document. The protocols for this study were approved by the Ethical Committee of the People's Hospital of Xinjiang Uygur Autonomous Region. All subsequent research analyses were carried out in accordance with the approved guidelines and regulations.

### 2.2 Dnadeoxyribonucleic acid extraction and relative telomere length measurement

Genomic DNA was isolated from whole-blood samples using the GoldMag-Mini Purification Kit (Gold-Mag Co. Ltd., Xi’an, People's Republic of China), and DNA concentrations were measured using the NanoDrop 2000 (Thermo Scientific, Waltham, MA). RTL was measured using the real-time quantitative polymerase chain reaction (PCR) method as described by Cawthon.^[17]^ Gene-specific amplification was performed in a ViiATM7 Dx Real-Time PCR Instrument (AB). The intra-assay or inter-assay differences were controlled by assaying each sample in 2 to 3 replicates or a calibrator DNA sample in different plates and the acceptable coefficient of variation (CV) was lower than 5% for cycle threshold values. 36B4 on chromosome 12, encoding acidic ribosomal phosphoprotein P_0_, was used as the single copy gene. All samples for both the telomere and 36B4 gene amplifications were always done in duplicate in separate 96- well plates. The cycle threshold is the number of cycles required for the fluorescent signal to cross the threshold. Ct values generated were used to calculate the telomere (T) repeat copy number to a single gene (S) copy number (T/S ratio) for each sample using the equation: T/S = 2^−(ΔCt)^, (ΔCt = Ct_telomere_ − Ct_36B4_). The relative ratio of T/S was defined as the ratio of each sample 2^−ΔCt^ to a calibrator DNA 2^−ΔCt^, 2^−(ΔΔCt)^. The primer sequences are shown in Supplementary Table S1.

### 2.3 Single-nucleotide polymorphism (SNP) selection and genotyping

In this study, 15 SNPs in *TERC*, *MYNN*, *NAF1*, *TNIP1*, *RTEL1*, *ZNF208* were selected from the 1000 Genomes Project (http://www.1000genomes.org/) for analysis and each had minor allele frequency >5% in Chinese Han population. The primers were designed online (https://agenacx.com/online-tools/). The PCR primers for each SNP are shown in Supplementary Table S2. Agena MassARRAY Assay Design 3.0 software was used to design a multiplexed SNP Mass EXTENDED assay. Genotyping was performed on an Agena MassARRAY RS1000 platform using the manufacturer's protocol. Data management and analysis were performed using the Agena Typer 4.0 Software.

### 2.4 Statistical analyses

Pearson test was used to examine differences of categorical variables between different groups. The chi-square test and the Welch *T* test was used to examine differences of categorical variables and continuous variables between cases and controls, respectively. Mann–Whitney *U* test was used for RTL comparison between different groups. To evaluate the association between RTL and GC risk, unconditional logistic regression was used to determine odds ratios (ORs) and 95% confidence intervals (CIs). The variable of age and gender were adjusted in multivariate unconditional logistic regression analysis in order to eliminate these residual confounding effects. Statistical analyses were performed using the Microsoft Excel (Microsoft Corp., Redmond, WA) and Statistical Package for the Social Sciences (SPSS) statistics 19.0 version software (SPSS Inc, Chicago, IL). *P* values <.05 were considered statistically significant.

Allele and genotype frequencies were determined using direct counts. SNP allele frequencies in the controls were tested for departure from Hardy–Weinberg Equilibrium (HWE) before analysis. Allele and genotype frequencies in GC patients and controls were calculated using chi-squared and Fisher exact tests. Associations between SNPs and the risk of GC were tested in genetic models using PLINK software (Version 1.07). Unconditional logistic regression analysis was used to examine the ORs and 95% CIs in order to assess the association between SNPs and GC risk. Four models (co-dominant, dominant, recessive, and log-additive) were used to test the association between SNPs and GC risk. Finally, the Haploview software package (version 4.2) and SHEsis software platform (http://shesisplus.bio-x.cn/SHEsis.html) were used to estimate pairwise linkage disequilibrium (LD), haplotype construction, and genetic association at polymorphism loci. All *P* values were 2-sided, and *P* < .05 indicates statistical significance.

## 3 Results

### 3.1 Association analysis of telomere length and risk of gastric carcinoma

A total of 1000 GC cases (532 males and 468 females) and 1100 healthy controls (514 males and 586 females) were included in this study. The epidemiological and clinical characteristics of the participants were summarized in Table 1. The ages of controls and cases were 63.69 ± 9.26 years and 62.51 ± 7.76 years (*P* > .05), respectively. There was no significant difference in either smoking status or drinking status between cases and controls (*P* > .05).

**Table 1:**
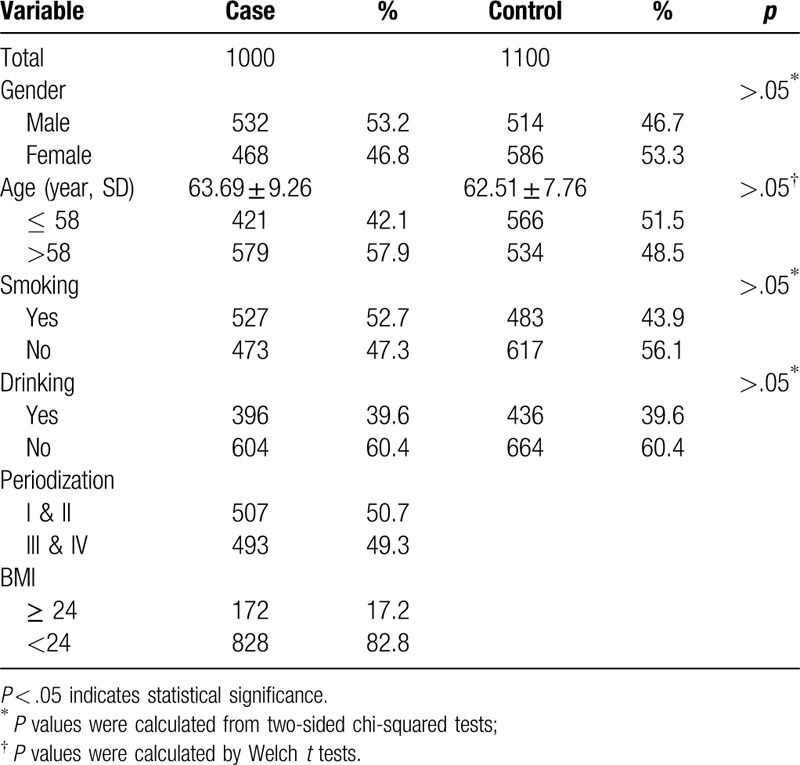
Basic characteristic of cases and controls.

We performed real-time quantitative PCR to measure the RTL of peripheral blood leukocytes (PBLs) from cases and controls. The mean inter-assay CV of real-time PCR reaction was 6.2% (range, 3.6%–9.5%), whereas intra-assay CV was 5.3% (range, 2.8%–7.1%). The results indicated that GC patients had notably shorter median RTL than healthy controls (0.83 vs 1.24; *P* < .001) (Table 2). When comparing RTL according to gender stratification, age of 58 years, smoking status and drinking status, Mann–Whitney *U* test showed that both groups of GC patients in male and female had statistically shorter median RTL than relevant healthy controls (0.81 vs 1.02, *P* < .001; 0.86 vs 1.26, *P* < .001). The analysis results indicate that both groups of GC patients in age ≤58 years and age >58 had statistically shorter median RTL than relevant healthy controls (0.81 vs 1.32, *P* < .001; 0.66 vs. 0.92, *P* < .001). We also found that the groups of GC patients in smoking and no-smoking or drinking and no-drinking had statistically shorter median RTL than relevant healthy controls.

**Table 2:**
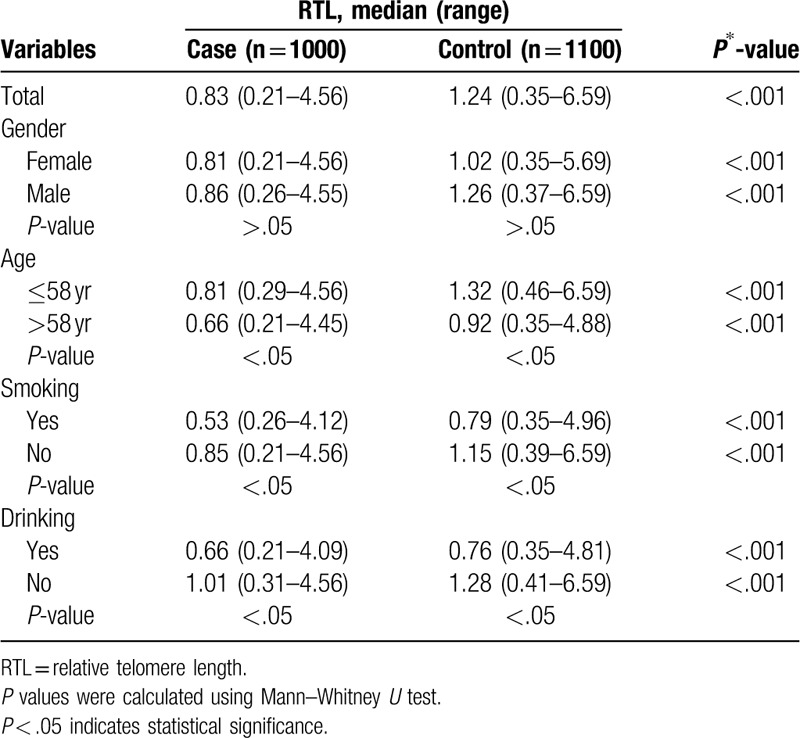
Distributions of RTL by host characteristic in all participants.

We performed an unconditional multivariate regression analysis to investigate the association between the RTL and GC risk. The participants were divided into 2 groups based on the median RTL, we observed that the shorter RTL (<0.8328) significantly increased risk of GC as compared with the longer RTL (≥0.8328) when adjusted by age, gender, smoking and drinking (OR = 12.67, 95% CI 8.96–20.45, *P* < .001) (Table 3). To explore whether age, sex, smoking, and drinking influenced the observed associations, we conducted stratified analyses by sex (male and female), age (≤58 years and >58 years), smoking status and drinking status for case-control samples as shown in Table 3. As compared with the longer RTL (≥0.8326), we observed that the shorter RTL (<0.8326) significantly increased risk of GC in male (OR = 8.94, 95% CI: 5.10–15.67, *P* < .001), female (OR = 7.71, 95% CI: 4.33–13.95, *P* < .05), age >58 years (OR = 9.91, 95% CI: 6.50–19.78, *P* < .001), smoking (OR = 4.88, 95% CI: 3.23–11.56, *P* < .001), no-smoking (OR = 6.14, 95% CI: 3.87–13.63, *P* < .05), drinking (OR = 5.55, 95% CI: 4.01–15.69, *P* < .001), and no-drinking (OR = 7.11, 95% CI: 3.68–14.04, *P* < .05), except for the age ≤58 years subjects (*P* > .05).

**Table 3:**
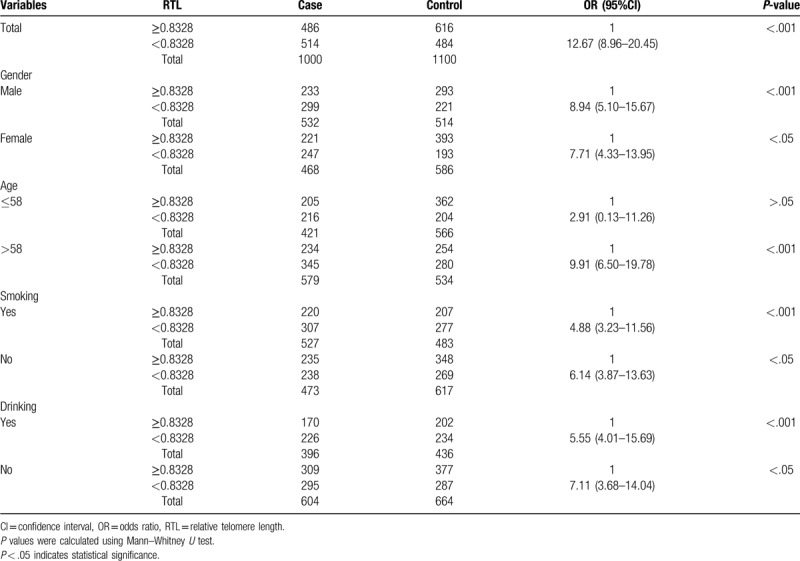
Stratified analysis of the association between the RTL and the risk of GC.

### 3.2 The association between telomere length-related genes polymorphisms and gastric carcinoma risk

Table 4 summarized the basic information of candidate SNPs in our study, such as chromosomal position, gene, allele, HWE test results, and minor allele frequency, 95% CI, and the *P* values. In control groups, all SNPs were in line with HWE (*P* > .05). Pearson chi-squared test was used to assess the associations between SNPs variants and the risk of GC in the allele models. We found that the SNPs rs10069690, rs2242652, and rs2853676 in the *TERT* were significantly associated with increased GC risk (rs10069690: OR = 1.33, 95% CI: 1.04–2.70, *P* = .0002; rs2242652: OR = 1.46, 95% CI: 1.28–2.92, *P* = .00042; rs2853676: OR = 2.04, 95% CI: 1.83–4.30, *P* = .0014). The other 2 SNPs rs7708392 and rs10036748 (in the *TNIP1*) were also associated with increased GC risk (rs7708392: OR = 1.65, 95% CI: 1.31–3.84, *P* = 3.5e–5; rs10036748: OR = 2.04, 95% CI: 1.83–4.31, *P* = 2.3e–5).

**Table 4:**
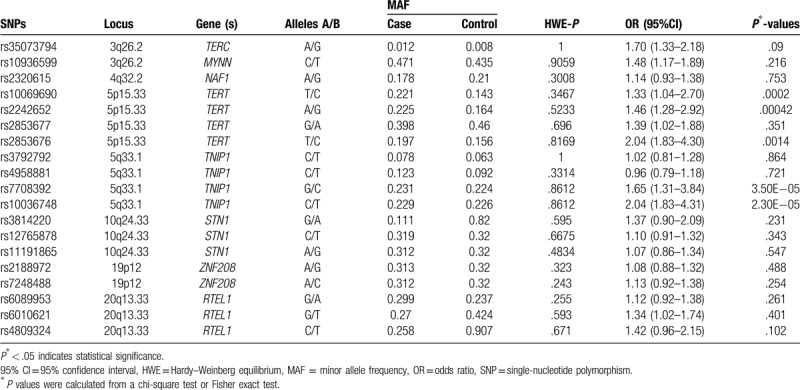
Allele frequencies in cases and controls and OR estimates for GC risk.

As is shown in Table 5, logistic regression analyses revealed that the rs6010620 (*TERT*) polymorphism conferred an increased risk of GC in the codominant model (OR = 1.82, 95% CI: 1.45–2.30, *P* = .013 for the "T/T” genotype), the dominant model (OR = 1.69, 95% CI: 1.47–2.43, *P* = .006 for the "C/T-T/T” genotype) and log-additive model (OR = 1.67, 95% CI: 1.19–2.18, *P* = .002), respectively. The rs2242652 (*TERT*) polymorphism was associated with increased risk of GC in the dominant model (OR = 2.05, 95% CI: 1.79–3.39, *P* = .026 for the "A/G-A/A” genotype) and log-additive model (adjusted: OR = 1.47, 95% CI: 1.24–2.29, *P* = .011), respectively. The rs2853676 (*TERT*) polymorphism was associated with increased risk of GC in the codominant model (OR = 1.89, 95% CI: 1.63–4.28, *P* = .008 for the “T/T” genotype), the dominant model (OR = 1.72, 95% CI: 1.36–2.60, *P* = .003 for the "C/T-T/T” genotype) and log-additive model (OR = 1.62, 95% CI: 1.19–2.21, *P* = .003), respectively. The rs7708392 (*TNIP1*) polymorphism was associated with increased risk of GC in the dominant model (OR = 1.44, 95% CI: 1.15–2.60, *P* = .032 for the "C/G-C/C” genotype). The rs10036748 (*TNIP1*) polymorphism was associated with increased risk of GC in the codominant model (OR = 1.75, 95% CI: 1.68–2.38, *P* = .004 for the "C/C” genotype), the dominant model (OR = 1.46, 95% CI: 1.25–2.74, *P* = .026 for the "C/T-C/C” genotype) and log-additive model (adjusted: OR = 1.96, 95% CI: 1.36–2.48, *P* = .029), respectively.

**Table 5:**
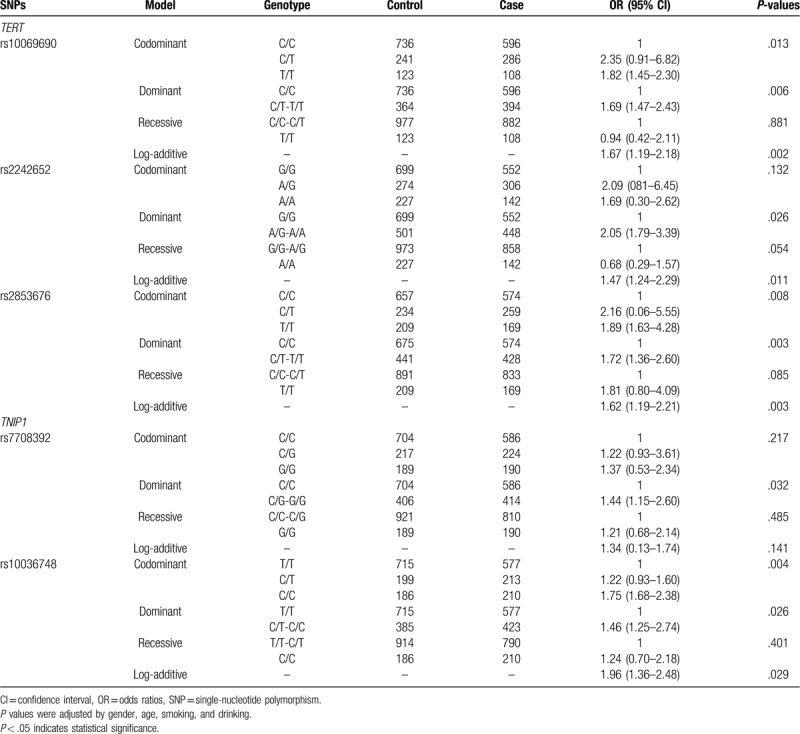
Association between candidate SNPs and the risk of GC under genotype models.

**Figure 1:**
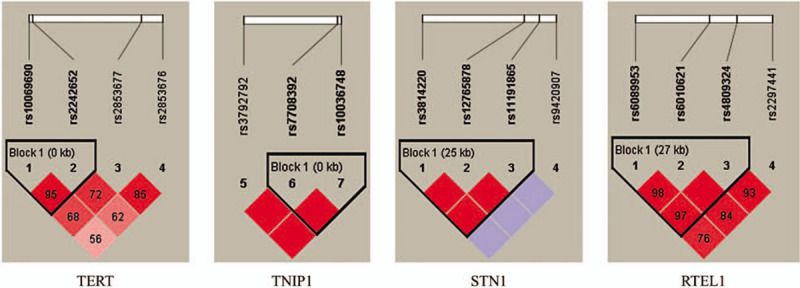
Haplotype block map for single-nucleotide polymorphisms in the *TERT*, *TNIP1*, *STN1*, and *RTEL1* genes.

Haploid blocks were obtained by Haploview4.2 software for haploid analysis of candidate SNP sites in control population. We observed that the SNPs rs10069690 and rs2242652 in the *TERT* had very strong linkage disequilibria, it forms 1 LD block. One block was detected in studied *TNIP1* SNPs (rs7708392 and rs10036748) by haplotype analyses. The SNPs (rs3814220, rs12765878, and rs11191865) on the *STN1* gene and the SNPs (rs6089953, rs6010621 and rs4809324) on the *RTEL1* gene formed 1 LD block, respectively (Fig. 1). Finally, the haplotypes with frequencies of more than 0.05 were selected for further research (Table 6). Haplotype analysis revealed the block in the *TERT* gene, the "GC” haplotype was associated with increased risk of GC (OR = 1.35, 95% CI: 1.03–1.78, *P* = .004) (Table 6). The association between the *TNIP1* haplotype and the risk of GC was shown in the Table 6. The result showed that the "G_rs7708392_T_rs10036748”_ haplotype was associated with increased the risk of GC (OR = 1.59, 95% CI: 1.18–0.94, *P* = .041).

**Table 6:**
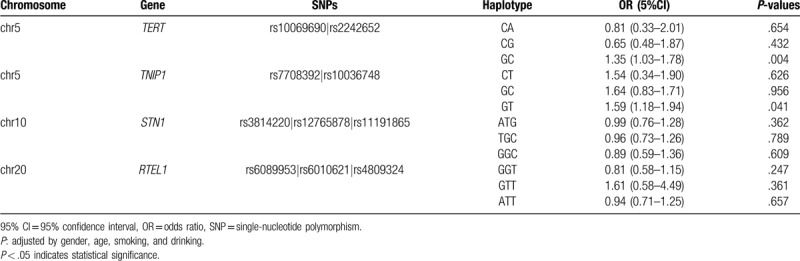
Haplotype analysis results of this study.

## 4 Discussion

Several studies showed that the etiology and pathogenesis of GC were likely to comprise a multifactorial disorder resulting from environmental and genetic factors and their interaction. In the present case–control study, we studied the role of RTL in susceptibility to GC and investigate the association between genetic polymorphisms in the telomere length related genes and GC risk. The results showed that the RTL in the case group was shorter than in the controls, and the shorter RTL was associated with increasing the risk of GC. In addition, smoking, drinking and different age range may also affect the telomere length. Association analysis between telomere length related genes polymorphisms and GC indicated that *TERT* (rs10069690, rs2242652, and rs2853676) and *TN1F1* (rs7708392 and rs10036748) were significantly increasing the risk of GC. The results indicated that the telomere length and the *TERT* and *TNIP1* genes may play important roles in GC risk in the Chinese population.

To date, many studies have examined telomere length in PBLs and its association with cancer risks.^[18–20]^ However, the results remain inconsistent with positive, negative, or null associations between telomere length and cancer risks. The majority studies have shown that short telomere length is significantly associated with increased risks of cancers such as breast cancer,^[21]^ papillary thyroid carcinoma,^[22]^ lymphoblastic leukemia,^[23]^ glioma,^[24]^ etc. On the contrary, longer telomere has also been found to be associated with increased risks of colorectal adenoma,^[25]^ prostate cancer,^[26]^ esophageal cancer,^[27]^ and renal cell carcinoma,^[28]^ etc. Interestingly, our findings indicate that the shorter RTL are associated with higher risk of GC, suggesting a significant association between RTL in PBLs and GC risk consistent with the report of Liu et al, who conducted a case-control study consisting of 524 gastric cardia adenocarcinoma (GCA) cases and 510 controls samples in Chinese Han population, the result indicated that short RTL was associated with increasing the susceptibility of GCA.^[29]^ In the meanwhile, another research reported that short leukocyte RTL significantly associated with poor prognosis of GC patients.^[30]^ In addition, the present study found that smoking, drinking and different age range may also be risk factors affecting the telomere length. These findings indicated that RTL might be a promising marker to identify high-risk individuals. Certainly, differences in study design, specific cancer site, limited statistical power, variability in confounding factors, and laboratory measurement of telomere length maybe contributing factors to these discrepancies.

In addition to the *TERT*, the *TERC* gene plays an important role in encoding the telomere RNA.^[31]^ The *STN1* gene is specifically involved in telomere replication and end sealing.^[32]^ The *NAF1* gene can change telomere length by affecting the level of telomerase RNA transcription.^[33]^ The *RTEL1* gene also plays an important role in the stability, protection, and elongation of telomeres.^[33]^ The *TNIP1* and *ZNF208* were identified by genome-wide association studies (GWAS) with affecting mean telomere length and their association diseases. Until now, many researches have reported that polymorphisms in these genes may affect the predisposition to telomere dysfunction-related malignancies, including GC.^[34–36]^ Zhang et al, found that *TERT* (rs10069690 and rs2853676) was significantly associated with increasing the GCA development.^[37]^ Zhang et al, found that the rs2736100 and rs2853669 in *TERT* gene were associated with increased GC risk.^[38]^ In the present study, we identified that the *TERT* (rs10069690, rs2242652 and rs2853676) was associated with increased risk of GC, which was consist with the report of Zhang et al. The current findings also suggested that the *TNIP1* (rs7708392 and rs10036748) can be considered as a risk factor for GC. However, we have not found the biological relevance between the polymorphisms of other telomere length related genes (*TERC*, *MYNN*, *NAF1*, *STN1*, *ZNF208*, and *RTEL1*) and GC risk. Until now, little research has been done on the correlation between *TERC*, *MYNN*, *NAF1*, *TNIPI*, *STN1*, *ZNF208*, and *RTEL1* gene polymorphism and GC risk.

To sum up, we provide new evidence for the association between RTL and RTL-related genes variants and GC risk in Chinese population for the first time, which may provide new data to facilitate earlier diagnosis and promote early prevention, and shed light on the new candidate genes and new ideas for the study. Nevertheless, there are limitations that need to be noticed. Our current research is fundamental, further studies in larger samples and biological functional assays are warranted to validate our findings.

## 5 Conclusion

The results indicated that the RTL in the case group was shorter than in the controls, and the shorter RTL was associated with increased risk of GC. The polymorphisms of *TERT* (rs10069690, rs2242652, and rs2853676) and *TNIP1*(rs7708392 and rs10036748) were significantly associated with increased GC risk.

## Acknowledgment

We thank all the patients and individuals for their participation. We thank the physicians and nurses of the People's Hospital of Xinjiang Uygur Autonomous Region for their offers of gastric carcinoma blood samples.

## Author contributions

**Data curation:** Fan Yuxiang.

**Formal analysis:** Xu Rong.

**Investigation:** Su Ying.

**Methodology:** Xu Rong.

**Project administration:** Chen Ru.

**Resources:** Han Zhongcheng, Chen Ru.

**Software:** Han Zhongcheng.

**Supervision:** Su Ying.

**Visualization:** Su Ying.

**Validation:** Jiang Liu.

**Writing – original draft:** Ma Lili.

**Writing – review & editing:** Fan Yuxiang, Jiang Liu.

## Supplementary Material

Supplemental Digital Content

## Supplementary Material

Supplemental Digital Content
