# Supplementary material for: Genetic variations associated with telomere length affect the risk of gastric carcinoma
Source: Medicine (Baltimore). 2020 Jun 5;99(23):e20551. doi: 10.1097/MD.0000000000020551 (PMC7306382; doi:10.1097/MD.0000000000020551)
Supplement: Supplemental Digital Content [file medi-99-e20551-s001.docx]

Supplementary table S1: Telomere amplification primer and 36B4 gene sequence

| Amplified gene | Primer sequences |
| --- | --- |
| Telomere | Tel-F: GGTTTTTGAGGGTGAGGGTGAGGGTGAGGGTGAGGGT |
|  | Tel-R: TCCCGACTATCCCTATCCCTATCCCTATCCCTATCCCTA |
| 36B4 | 36B4-F: cag caa gtg gga agg tgt aat cc |
|  | 36B4-F: ccc att cta tca tca acg ggt aca a |
