# Supplementary material for: Genetic variations associated with telomere length affect the risk of gastric carcinoma
Source: Medicine (Baltimore). 2020 Jun 5;99(23):e20551. doi: 10.1097/MD.0000000000020551 (PMC7306382; doi:10.1097/MD.0000000000020551)
Supplement: Supplemental Digital Content [file medi-99-e20551-s002.docx]

Supplementary table S2: The PCR primers of each SNPs

| locus | 1st-PCR primer | 2nd-PCR | UEP sequences |
| --- | --- | --- | --- |
| rs35073794 | ACGTTGGATGGTCTTCCGCTTTTTGTTGCC | ACGTTGGATGAGAAGCAAAAACCTCAACA | cctCAAAAACCTCAACAAAATCT |
| rs10936599 | ACGTTGGATGTTCCCGCTGTTTGTTCAGTC | ACGTTGGATGCAAGGGTAAAATTCCATTCTG | ATGCAGTATTCGCACCA |
| rs2320615 | ACGTTGGATGACCAATTTAACAAGACAGC | ACGTTGGATGAGGCAGAGACATTCCATTTG | CCATTTGAAAAGAAATAATTCTACT |
| rs10069690 | ACGTTGGATGCCTGTGGCTGCGGTGGCTG | ACGTTGGATGATGTGTGTTGCACACGGGAT | GGGATCCTCATGCCA |
| rs2242652 | ACGTTGGATGACAGCAGGACACGGATCCAG | ACGTTGGATGAGGCTCTGAGGACCACAAGA | gtcgGAGGACCACAAGAAGCAGC |
| rs2853677 | ACGTTGGATGATCCAGTCTGACAGTCGTTG | ACGTTGGATGGCAAGTGGAGAATCAGAGTG | gggtAATCAGAGTGCACCAG |
| rs2853676 | ACGTTGGATGTGTCTCCTGCTCTGAGACC | ACGTTGGATGCAAAACTAAGACCCAAGAGG | agatGGAAGTCTGACGAAGGC |
| rs3792792 | ACGTTGGATGCTCAGATCAGTTCACTCCTC | ACGTTGGATGATGGCAGCTGTTACGGCCAC | ccctTTACGGCCACCACCAAGCATG |
| rs4958881 | ACGTTGGATGCACAAATATGTGGACAGTTT | ACGTTGGATGTGCAATTCCACCCAAGGATG | GGATGAAAGGAAGTGAGA |
| rs7708392 | ACGTTGGATGAGGCCAACTGGTCAATTCTC | ACGTTGGATGGGGTCTCTTCTGGAACTTAG | ggggaTGGAACTTAGTAGACTAGTCA |
| rs10036748 | ACGTTGGATGGCAAAGCAGCCCCTTTTTTC | ACGTTGGATGCTTTCATAGCATGATACACG | ACGTATGAGAAAAATAAAATAGTAA |
| rs3814220 | ACGTTGGATGTGCTTGGCTTTTCATGAGGG | ACGTTGGATGAAGGACTGTGTTGGAACCTG | ACCCCGCTTCATAATGT |
| rs12765878 | ACGTTGGATGACATTGCCTTGTACAACTCC | ACGTTGGATGCCACAAAATATCTAGCTTGGG | AGCTTGGGCTCTTGA |
| rs11191865 | ACGTTGGATGCTTCTCCCCATCTGAAAGTG | ACGTTGGATGGGATTCTTTGGGTCTCCAAG | cccaaAGATCCTAGCATATACCACT |
| rs2188972 | ACGTTGGATGGGCTTGATTGGTCAAATGGC | ACGTTGGATGATTCAGAACCTGTGCAAAGC | GACTTCTCAAAGAACTAGAAA |
| rs7248488 | ACGTTGGATGGTTCTCCAGGAACACTTATG | ACGTTGGATGGCAGAGTGTTTTCCTGGTTG | GTCATGATGAGAAGGGT |
| rs6089953 | ACGTTGGATGCCCTTCAAAGGACGATCGTT | ACGTTGGATGGCGTCTGTCATAAAAAGGGC | ggGTTCCAGGTGGGGTC |
| rs6010621 | ACGTTGGATGACCCCATCCCTCCCCTCTGA | ACGTTGGATGAGCACGAGAACAGCACCGAG | ACAGCACCGAGGAAAAG |
| rs4809324 | ACGTTGGATGAGCCGGTGCACAGATTCCAA | ACGTTGGATGGAGAAGTCAAGTGACATCAG | gTCAGAGGTCAATGGAACA |
